# Tuberculous lymphadenitis ‐ A case report

**DOI:** 10.1002/ccr3.5198

**Published:** 2021-12-13

**Authors:** Filipa Leal, Francisco Pombo, Ana Silva Rocha

**Affiliations:** ^1^ Department of Internal Medicine Centro Hospitalar do Tâmega e Sousa Guilhufe Portugal

**Keywords:** extrapulmonary, lymphadenitis, scrofula, tuberculosis

## Abstract

Although tuberculosis manifests mostly as a pulmonary disease, extrapulmonary presentations can occur and must be taken into consideration depending on the clinical setting.

## Graphical abstract

Despite the decrease in the incidence of tuberculosis in developed countries, it still poses an important health issue. Manifestations can be very variable therefore, clinical suspicion is essential for a precocious diagnosis.

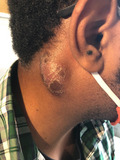

## 1 CASE DESCRIPTION

**FIGURE 1:**
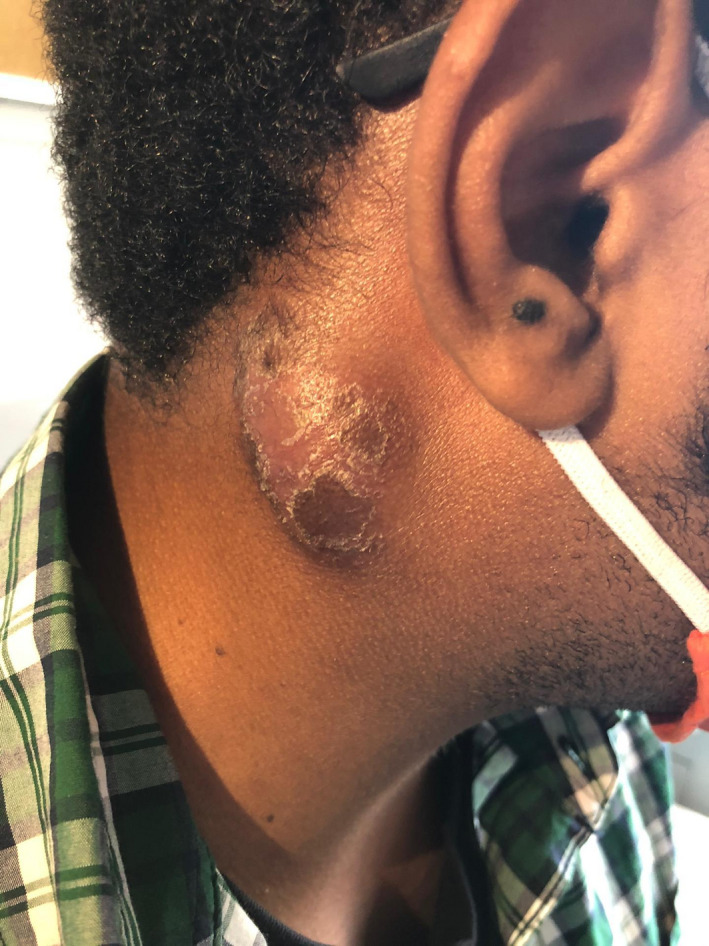
Right retroauricular mass

A 25‐year‐old Angolan male patient presented with 2‐month history of asthenia, weight loss, and cervical swelling. Examination showed a right 5‐cm retroauricular firm mass (Figure 1).

Blood workup showed no anemia, mild lymphopenia, elevated C‐reactive protein (22 mg/L), and sedimentation rate (53 mm). HIV infection was ruled out, and the interferon‐gamma release assay was positive. A biopsy was performed and revealed necrotizing granulomatous lymphadenitis suspicious of tuberculous lymphadenitis. Both protein chain reaction and cultures for the detection of *Mycobacterium tuberculosis* were positive. The diagnosis of tuberculous lymphadenitis (A.K.A. scrofula) was made, and the patient was started on isoniazid, rifampin, pyrazinamide, and ethambutol for the first 2 months, followed by isoniazid and rifampin for an additional 4 months, with a total of 6 months treatment, with good tolerance and favorable response.

Despite consistent decrease in tuberculosis (TB) incidence in the World Health Organization European regions, it still poses a major public health issue.^1^ *Mycobacterium tuberculosis* infections manifests mostly as pulmonary disease, but extrapulmonary manifestations can occur.^2^

Extrapulmonary TB is not always easy to diagnose, and in cases like ours, biopsy of the lymph node, with TB smear and culture exams, is critical to the diagnosis. This case highlights the importance of precocious recognition and treatment to prevent further complications.

## CONFLICT OF INTEREST

There are no conflicts of interest.

## AUTHOR CONTRIBUTIONS

Author 1: Involved in patient care, data and information collection and main author of the manuscript. Author 2: Involved in patient care and manuscript review. Author 3: Involved in patient care and manuscript review.

## ETHICAL APPROVAL

The authors have no ethical conflicts to disclose.

## CONSENT

Written informed consent was obtained from the patient to publish this report in accordance with the journal's patient consent policy.

## Data Availability

Data sharing is not applicable—no new data generated.
